# Testosterone therapy as a novel approach to the management of cytopenias in myelodysplastic neoplasms: a review of literature and case report

**DOI:** 10.1007/s00432-024-05844-w

**Published:** 2024-08-29

**Authors:** John Yan, Bradley Rockwell, Divij Verma, Srabani Sahu, Mendel Goldfinger, Amit Verma

**Affiliations:** 1 Department of Internal Medicine, Montefiore Medical Center, Bronx, NY USA; 2 Division of Hemato-Oncology, Blood Cancer Institute, Montefiore Einstein Comprehensive Cancer Center, Bronx, NY USA; 3 Division of Oncology, Albert Einstein College of Medicine, 111 East 210 Street, Bronx, NY 10467 USA

**Keywords:** Myelodysplastic neoplasm, Testosterone, Cytopenia, Anemia, Hypogonadism

## Abstract

**Purpose:**

To explore the potential of testosterone therapy in managing cytopenias in myelodysplastic neoplasm and investigate the link between hypogonadism and hematologic malignancies.

**Methods:**

A case of a patient with intermediate-risk myelodysplastic neoplasm and hypogonadism treated with testosterone replacement therapy is presented. Testosterone, prostate specific antigen, and erythropoietin levels were checked prior to therapy initiation and 3 months after. Blood counts were monitored over time. This is followed by a literature review of testosterone use in myelodysplastic neoplasm and the prevalence of hypogonadism in hematologic malignancies.

**Results:**

The patient showed sustained improvement in anemia with testosterone therapy and reported subjective improvement in his weakness and fatigue. This improvement occurred even in the setting of an undetectable follow up erythropoietin level. His repeat prostate specific antigen levels remained low, while testosterone levels showed marked improvement. The literature review demonstrated positive response rates for testosterone in treating myelodysplastic neoplasm-related cytopenias, and showed a higher incidence of hypogonadism in hematologic malignancies.

**Conclusion:**

Our review suggests that the use of testosterone in low and intermediate-risk myelodysplastic neoplasm is underexplored and poses to have significant potential as a future therapeutic agent, after careful consideration of risks and benefits. In addition, the incidence of hypogonadism in myelodysplastic neoplasm and its potential impact on exacerbating cytopenias in myelodysplastic neoplasm warrants further investigation.

## Introduction

Myelodysplastic neoplasm (MDS) represents a heterogeneous group of clonal hematopoietic disorders of which anemia is a common clinical manifestation that poses a major therapeutic challenge (Tefferi et al. 2009). Anemia and transfusion dependency are both associated with worsened survival and have significant impact on outcomes (Malcovati et al. 2006; Oliva et al. 2012). The management of anemia in low- to intermediate-risk MDS has traditionally focused on therapeutics aimed at minimizing red blood cell (RBC) transfusion burden. Current approaches include the use of erythropoiesis-stimulating agents (ESAs), synthetic steroids, hypomethylating agents, luspatercept, lenalidomide, and immunosuppressive agents (Lewis et al. 2021). Unfortunately, many patients are or become refractory despite advances in treatment, highlighting the need for alternative agents. Testosterone, known to possess erythropoietic properties (Moriyama et al. 1975), is underexplored as a potentially novel approach to treating MDS-related anemia. To date, very few studies have investigated the possible impact of testosterone replacement therapy (TRT) in MDS patients, especially in the context of hypogonadism, a prevalent condition in elderly patients (Mulligan et al. 2006; Ferrucci et al. 2006). This report describes a 49-year-old male with intermediate-risk MDS and hypogonadism who experienced improvement in anemia after treatment with aranesp and intramuscular (IM) testosterone, followed by a comprehensive literature review of testosterone treatment in MDS.

## Case presentation

A 49-year-old male with past medical history of chronic kidney disease stage 5 (CKD), adrenal insufficiency (on hydrocortisone), hypertension, trauma after a fall, and gout presented to oncology clinic early November 2023 for management of newly diagnosed intermediate-risk MDS after he was found to be pancytopenic during an inpatient admission for hematuria due to foley trauma and acute kidney injury on CKD. His blood counts were notable for macrocytic anemia with hemoglobin (hgb) 6.7 g/dL, MCV 101 fL, platelets (plt) 35 K/uL, and absolute neutrophil count (ANC) 1.3 K/uL. Bone marrow biopsy confirmed MDS, while next-generation sequencing noted STAG2, KRAS-G12D, and CHEK2 mutations. Cytogenetics showed normal male karyotype and flow cytometry did not note any increase in blasts. The patient was transfused 1 unit of packed RBCs and 1 unit of platelets during admission. Additional work up noted inappropriately low reticulocyte count (26.3 K/uL) and iron deficiency (iron 25 ug/dL, ferritin 228.0 ng/mL) for which he received a course of IV iron. His hematuria self-resolved mid-admission and was thought to be a negligible component of his persistent anemia.

Upon presentation to the clinic, the patient reported significant fatigue, generalized weakness, and difficulty with erections. Lab work showed hgb 8.9 g/dL, plt 75 K/uL, ANC 3.1 K/uL, low erythropoietin (EPO) (5.9 mU/mL) and low total testosterone (TT) (198 ng/dL). Additional workups including vitamin B12, folate, TSH, reticulocyte count, and iron studies were all unremarkable. Per endocrine evaluation, the patient was diagnosed with hypogonadotropic hypogonadism due to chronic opioid use for pain relief. The patient was subsequently started on aranesp 300 MCG infusion every 3 weeks (first dose 11/8/2023) and bi-weekly testosterone cypionate 200 mg/mL injections (first dose 11/27/2023). Initial Prostate serum antigen (PSA) was within normal limits (1.2 ng/mL).

**Fig. 1:**
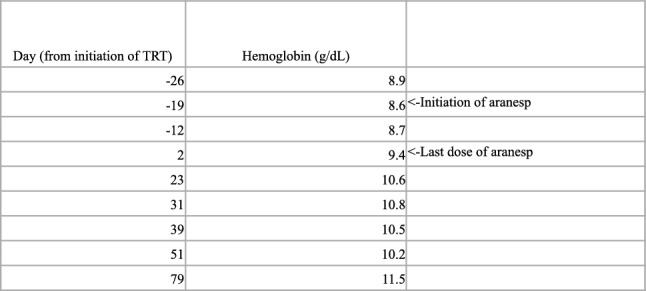
Hemoglobin trend since first clinic presentation and initiation of testosterone enanthate and aranesp

At his one-month follow-up after having received 2 doses of aranesp and testosterone respectively, he endorsed significant improvement in his fatigue and weakness. His hgb was improved to 10.6 g/dL, and further aranesp was deferred. By February 2024, nearly 3 months later, his HGB had improved to 11.5 g/dL while on testosterone monotherapy (ref Fig. 1). Notably, repeat EPO level was below detectable levels (< 5 mU/mL) while repeat TT level improved to 574 ng/dL. Repeat PSA (1.3 ng/mL), reticulocyte, and iron studies all remained within normal limits, and his plt level remained stable (75 K/uL). The patient continued to deny any fatigue, and even endorsed increases in appetite and weight. No adverse events were noted throughout the duration of his treatments. Informed consent was obtained from the patient for the publishing of this report.

## Discussion and review

We present a case where a patient with intermediate-risk MDS and hypogonadism experienced a sustained increase in hgb levels of over 2 g/dL using testosterone and ESAs. The role of testosterone in enhancing erythropoiesis is widely recognized in laboratory and clinical settings, and TRT has well documented use in treating anemia for a variety of conditions such as aplastic anemia, end-stage renal disease, and hypogonadism (Pencina et al. 2023; Nassani et al. 2023; Ballal et al. 1991). While the mechanisms of its erythropoietic effect are still not fully understood, several hypotheses have been proposed, including stimulating EPO production, lowering hepcidin levels, prolonging RBC survival, and even directly influencing hematopoietic stem cells via IGF-1 induction (Moriyama et al. 1975; Guo et al. 2013; Beran et al. 1982). There is also clinical evidence to suggest that testosterone and ESAs are synergistic and should be used in combination for optimal effect (Ballal et al. 1991). Notably, our patient’s anemia continued to improve despite having low follow-up levels of EPO upon discontinuation of aranesp.

### Literature review of testosterone replacement therapy in MDS

The literature on testosterone in treating MDS-related anemia is scarce, with only two prospective studies and a single case report available (Table 1). Riccardi et al. (1987) in 1987 treated 101 patients with refractory cytopenia, 64 of whom had MDS, with high-dose testosterone enanthate (TE) (7–10 mg weekly IM). In the MDS cohort, 44% responded to TE, 94% of which had increase in hgb and/or reduction in transfusion requirement. Median duration of response was 13 months and responsive patients had improved survival versus non-responders. No pre-treatment testosterone levels were recorded.

**Table 1:** Summary of literature regarding testosterone in MDS

Author, year of publication	Study type	Cohort size	Intervention	Follow up period	Primary outcomes	Response features
Riccardi et al. (1987)	Prospective	45^a^	Testosterone enanthate IM (7–10 mg/kg) weekly	Median 37.9 months (range 6–174)	1) increase in hgb at least 3 g/dL or 50% decrease in transfusion requirement 2) doubling of granulocyte number 3) 1.5 × increase in plt number	-44% response, of which 94% had increase in hgb and/or reduction in transfusion requirement -median duration of response was 13 months
Mei et al. (2023)	Prospective	43	EPO (10000 IU/day) plus ATRA (25 mg/m^2^/day) and testosterone undecanoate (80 mg twice daily)	3 months	Increase in hgb ≥ 1.5 g/dL from baseline and transfusion independency	-65.1% response -no difference in response for EPO ≤ 500 vs > 500 -better response in SF3B1 (12/15, 80%) and worse in ASXL1 (3/8, 37.5%)
Iijima et al. (2011)	Case report	1	Testosterone enanthate 250 mg IM every 3 weeks	6 months	N/A	-improvement in hgb from 11.7 to 15.2 g/dL -improvement in plt from 23.6 to 38.3 K/µL

^a^Only inclusive of MDS patients who were evaluated for treatment response

Mei et al. (2023) observed a 65% response rate within 43 low-risk MDS patients who received a combination of EPO (10,000 IU/day), all-trans retinoic acid (25 mg/m^2^/day), and testosterone undecanoate (80 mg twice daily). Responders had an increase in hgb (greater than 1.5 mg/dL increase from baseline) and were transfusion independent. No significant difference in response was found between those with initial EPO ≤ 500 mU/mL versus > 500 mU/mL. Interestingly, better efficacy was noted in patients with SF3B1 compared to those with ASXL1 mutations. The most common adverse events reported were dry skin and fatigue. This study also did not report testosterone levels.

Finally, a case study by Iijima et al. (2011) detailed a 70-year-old man with low-risk MDS and hypogonadism who received TE and showed a sustained response in both his anemia and thrombocytopenia despite having previously failed the derivative synthetic testosterone methenolone in the past.

The limited studies reviewed vary in level of evidence and type of TRT used but overall suggest that TRT shows promise as a potential therapy for MDS-related cytopenia. Riccardi et al. and Mei et al. interestingly did not assess pre-treatment testosterone levels, and yet still reported substantial response rates in their cohorts. The above findings also hint at a potential association between genetic markers and efficacy of TRT, in addition to the possibility that TRT remains a viable alternative even in situations where anabolic-adrenergic steroids have not worked.

### Hypogonadism and MDS

Ferrucci et al. (2006) reported that male patients with lower TT had significantly reduced hgb, higher prevalence of anemia, and increased risk of developing anemia compared to subjects with normal TT. In anemic hypogonadal patients who received TRT, the TRAVERSE randomized control trial demonstrated a significant response in anemia and energy levels lasting up to 4 years (Pencina et al. 2023). The Hypogonadism in Males (HIM) study identified prevalence of hypogonadism in men aged ≥ 45 to be 38.7%, based on TT < 300 ng/dL (Mulligan et al. 2006). And while direct studies on hypogonadism incidence in MDS are lacking, studies in other malignancies report increased prevalence of hypogonadism when compared to healthy male cohorts. For example, a cross-sectional study by Fleishman et al. reported a 46% prevalence in leukemia and lymphoma, while a prospective study in multiple myeloma found a prevalence of hypogonadism of 74% (Fleishman et al. 2010; John et al. 2019).

### Adverse effects of TRT

The long-term safety of TRT, particularly regarding its links to prostate cancer and cardiovascular disease, is not well established. While there is evidence for the role of androgens in prostate cancer pathogenesis and TRT has been shown to increase PSA levels in some men, clinical studies have not demonstrated a significant increase in prostate cancer incidence or mortality in TRT-treated hypogonadal patients (Fernández-Balsells et al. 2010; Grech et al. 2014). Similarly, TRT has also not been conclusively linked to increased risk of myocardial infarction, stroke, venous thromboembolism, or overall mortality (Shores et al. 2021). However, it is also important to recognize that in the older MDS population, where median age of diagnosis is 72 years old and median survival is 5.3 years for low-risk cases (Tefferi et al. 2009), any theoretical mortality risk from prostate cancer or cardiovascular events is likely lower and possibly outweighed by the potential quality of life benefits of TRT.

## Conclusion

The potential role of TRT in managing MDS-related cytopenias, particularly in low- and intermediate-risk disease, is underexplored and could impact future therapeutic approaches. Additionally, routine testing of testosterone in MDS is currently not a widely adopted practice, and based on the available data it seems likely that we are significantly underdiagnosing and underestimating the role of hypogonadism in MDS anemia. Our case contributes to the ongoing broader discussion and available literature on the viability of testosterone treatment in MDS patients. Further research to investigate the incidence of hypogonadism in MDS and the efficacy and safety of TRT is necessary.

## Data Availability

The data from this study is available on reasonable request via contacting the corresponding author Dr. Amit Verma at amit.verma@einsteinmed.edu.
